# Computed Tomography Images of Spontaneous Portosystemic Shunt in Liver Cirrhosis

**DOI:** 10.1155/2022/3231144

**Published:** 2022-06-08

**Authors:** Fangfang Yi, Xiaozhong Guo, Qing-Lei Zeng, Benqiang Yang, Yanglan He, Shanshan Yuan, Ankur Arora, Xingshun Qi

**Affiliations:** ^1^Liver Cirrhosis Study Group, Department of Gastroenterology, General Hospital of Northern Theater Command (formerly called General Hospital of Shenyang Military Area), Shenyang 110840, China; ^2^Department of Infectious Diseases, The First Affiliated Hospital of Wenzhou Medical University, Zhejiang Provincial Key Laboratory for Accurate Diagnosis and Treatment of Chronic Liver Diseases, Wenzhou 325006, China; ^3^Department of Infectious Diseases and Hepatology, The First Affiliated Hospital of Zhengzhou University, Zhengzhou 450052, Henan, China; ^4^Department of Radiology, General Hospital of Northern Theater Command, Shenyang 110840, China; ^5^Postgraduate College, China Medical University, Shenyang 110122, China; ^6^Department of Gastroenterology, Xi'an Central Hospital, Xi'an 710003, China; ^7^Department of Radiology, Royal Liverpool University Hospital, Liverpool University Hospitals NHS Foundation Trust, Liverpool, UK

## Abstract

Spontaneous portosystemic shunt (SPSS) refers to collateral vessels that communicate between the portal vein system and systemic circulation. SPSS mainly includes esophageal varices, gastric varices, left gastric vein, recanalized paraumbilical vein, abdominal wall varices, and spontaneous splenorenal shunt. SPSS contributes to the development of hepatic encephalopathy caused by portal vein inflow bypassing and carries a higher risk of death in liver cirrhosis. Abdominal contrast-enhanced computed tomography is a major imaging approach to establish a diagnosis of SPSS and evaluate its location and feature. This review primarily describes the main contrast-enhanced CT features of SPSS in liver cirrhosis.

## 1. Introduction

**Figure 1:**
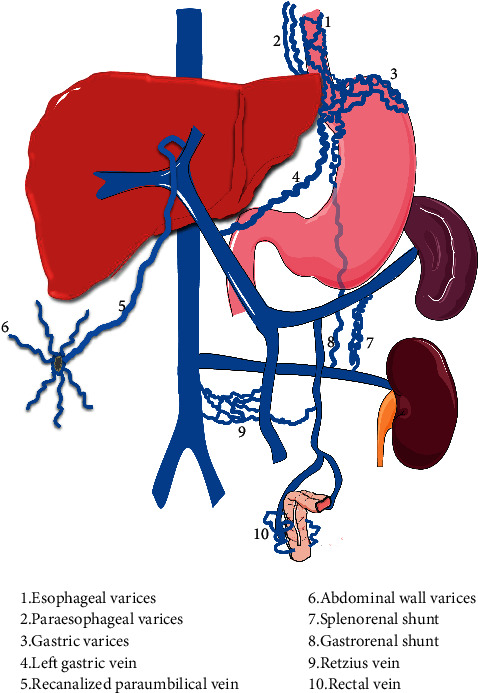
A schematic diagram of spontaneous portosystemic shunt in liver cirrhosis. Note: (1) esophageal varices; (2) paraesophageal varices; (3) gastric varices; (4) left gastric vein; (5) recanalized paraumbilical vein; (6) abdominal wall varices; (7) splenorenal shunt; (8) gastrorenal shunt; (9) Retzius vein; (10) rectal vein.

Spontaneous portosystemic shunt (SPSS) refers to collateral vessels that communicate between the portal vein system and systemic circulation [1] (Figure 1). SPSS is traditionally considered as a compensatory mechanism of portal hypertension in liver cirrhosis because it can decompress the portal venous system [2]. However, SPSS has been recently regarded as a feature of severe portal hypertension and associated with poor prognosis [3].

Until now, the pathophysiological mechanism of SPSS has not been completely elucidated. Traditionally, the formation of SPSS may be attributed to the dilatation of preexisting vessels, but recent studies suggest that it may also be related to neovascularization driven by vascular endothelial growth factor [4, 5]. Additionally, it seems that nonviral liver cirrhosis, especially alcoholic cirrhosis, is significantly associated with the development of SPSS [6, 7].

SPSS mainly includes esophageal varices, gastric varices, left gastric vein, recanalized paraumbilical vein, abdominal wall varices, and spontaneous splenorenal shunt (SSRS) [8]. SPSS contributes to a variety of serious complications, including gastrointestinal bleeding secondary to gastroesophageal variceal rupture and hepatic encephalopathy caused by portal vein inflow bypassing [1], and increases the risk of death [9].

A detailed description of the vascular anatomy of SPSS by imaging examinations is helpful to further understand the formation of collateral vessels secondary to portal hypertension. In recent years, abdominal contrast-enhanced computed tomography (CT) scan is a commonly used imaging technique to evaluate the development of collateral vessels [10]. Herein, we briefly review the prevalence, classification, clinical significance, and therapeutic implications of SPSS with an emphasis on the main contrast-enhanced CT features of SPSS in liver cirrhosis.

## 2. Prevalence

**Figure 2:**
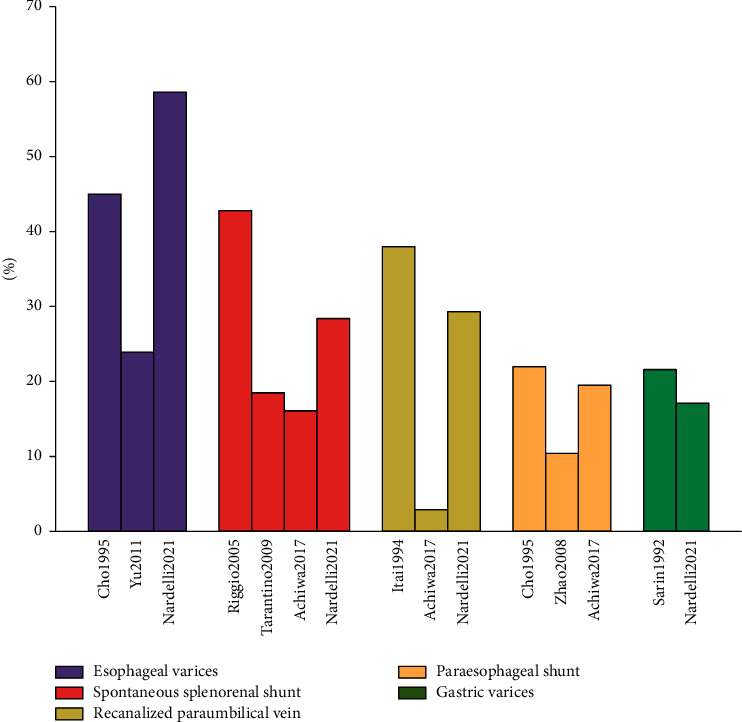
Prevalence of various types of SPSS on the contrast-enhanced CT scan.

The prevalence of various types of SPSS on the contrast-enhanced CT scan is different (Figure 2). Esophageal varices are one of the most common types of SPSS in cirrhotic patients undergoing CT scan, with a prevalence of 23.9–58.6% [11–13]. The prevalence of SSRS is 16.1–42.8% [10, 12, 14, 15], that of recanalized paraumbilical vein is 2.9–38% [12, 14], that of paraesophageal shunt is 10.4–22% [13, 14, 16], and that of gastric varices is 11.7–21.6% [12, 17].

## 3. Classifications

Traditionally, SPSS is termed as varices and shunts [18]. For example, gastroesophageal varices (GEVs) are attributed to the former type and SSRS to the latter one. Nowadays, SPSS is often classified as a drainage into the superior or inferior vena cava according to the outflow tract of collateral vessels [19]. Esophageal varices, paraesophageal varices, and gastric varices drain into the superior vena cava; by comparison, gastrorenal shunt, splenorenal shunt, recanalized paraumbilical vein, and abdominal wall varices drain into the inferior vena cava. It is also divided into small (i.e., the maximum diameter was <8 mm) and large (i.e., the maximum diameter was ≥8 mm) SPSS according to the maximum diameter of SPSS.

## 4. Clinical Significance

SPSS may be closely associated with the development of hepatic decompensation events, including hepatic encephalopathy, gastrointestinal bleeding, ascites, and portal vein thrombosis, and death (Table 1). First, large SPSS is a well-known precipitating factor for hepatic encephalopathy. Studies demonstrated that a maximum diameter of SPSS ≥ 8 mm or a total cross-sectional area of SPSS > 83 mm^2^ is more prone to develop hepatic encephalopathy [1, 7]. Second, the effect of SPSS on the development of gastrointestinal bleeding in liver cirrhosis remains controversial. Qi et al. found that cirrhotic patients with SSRS had a low prevalence of acute upper gastrointestinal bleeding, probably because it reduces portal pressure, thereby preventing the development of variceal bleeding [20]. By contrast, Nardelli et al. found that cirrhotic patients with SPSS had a higher risk of gastrointestinal bleeding and considered SPSS as a feature of severe portal hypertension and a marker of poor outcome [12]. Third, the association of SPSS with ascites in cirrhotic patients is also unclear. Renzulli et al. found that ascites was the most common decompensation event during follow-up in cirrhotic patients with SPSS and was an independent predictor of decompensation-free survival [25]. By contrast, Saks et al. reported that SSRS was associated with a lower risk of developing ascites in liver transplantation candidates, and the presence of ascites could not predict the risk of death in such patients [22]. Fourth, SPSS is associated with a higher risk of developing portal vein thrombosis in general patients with liver cirrhosis and liver transplantation recipients, probably due to its secondary reduction in portal blood flow [6, 9, 24]. Fifth, nearly all recent studies have found that SPSS had a lower transplantation-free survival and was an independent predictor for death in liver cirrhosis [1, 6, 7, 12].

## 5. Therapeutical Implications

Effective treatment of SPSS should be critical for improving the outcomes of cirrhotic patients and liver transplantation recipients. First, the rupture of GEVs can cause massive upper gastrointestinal bleeding and even lethal hemorrhagic shock [26]. Undoubtedly, the prophylaxis of high-risk GEVs from bleeding and urgent hemostasis of GEVs by vasoactive drugs, endoscopy, and/or transjugular intrahepatic portosystemic shunts (TIPS) should be necessary for saving the patients' lives [27, 28]. Second, large SPSS can induce refractory and recurrent hepatic encephalopathy, negatively influencing the quality of life and survival [7]. Thus, their closure by balloon-occluded retrograde transvenous obliteration (BRTO) and other vascular interventional treatments should be warranted for improving the patients' outcomes [29]. Third, TIPS can effectively decompress the portosystemic pressure and is mainly indicated for the treatment and prevention of GEVs' bleeding [27]. If adjunctive variceal embolization was performed during TIPS procedures, the risk of variceal rebleeding can be further decreased [30]. On the other hand, the presence of large SPSS increases the risk of post-TIPS overt hepatic encephalopathy [21]. Recently, a randomized controlled trial demonstrates that prophylactic embolization of large SPSS during TIPS procedures can reduce the risk of overt hepatic encephalopathy without any increased risk of other liver-related complications [31, 32]. These findings supported embolization of varices and SPSS during TIPS procedures. Fourth, SPSS has a portal blood flow “stealing” effect, reducing the portal blood flow into the graft and impairing the functional recovery of the graft, which may significantly affect the outcomes of liver transplant recipients [2]. Thus, it has been recommended that SPSS should be ligated during liver transplantation procedures [33].

## 6. Evaluation of SPSS on CT

Angiography is the gold standard diagnostic method used to examine the presence of SPSS, but invasive and expensive, even risky for patients with severe liver dysfunction [34]. By comparison, both contrast-enhanced CT and magnetic resonance imaging (MRI) scans are more convenient imaging methods. Notably, Renzulli et al. demonstrated excellent intraobserver and interobserver agreement in almost all types of SPSS detection and measurement by using CT [25]. Besides, identification of anatomical structures and characteristics (e.g., diameter) of SPSS by using CT can further strengthen the performance of currently available approaches for risk stratification in cirrhotic patients with complications of portal hypertension [35]. Except for measurement of the diameter of SPSS by CT, Praktiknjo et al. developed a software that could automatically calculate the cross-sectional area of SPSS based on the CT image processing [36] and found that the total cross-sectional area of SPSS was more advantageous than the diameter of SPSS for predicting the progression of cirrhosis [1].

As for the diagnosis of GEVs, endoscopy is the gold standard diagnostic method, and it can provide variceal eradication [37]. Contrast-enhanced CT and MRI scans are alternatives with excellent diagnostic accuracy for varices in liver cirrhosis [38–40]. Lipp et al. evaluated the effectiveness of CT and/or MRI examinations for the detection of esophageal varices compared with endoscopy and found that CT was a superior imaging method to MRI for the detection of esophageal varices and could accurately exclude the possibility of large esophageal varices to avoid the need or frequency of endoscopy screening in liver cirrhosis [41]. It should be noted that endoscopy fails to evaluate the entire spectrum of extraparietal GEVs and nongastroesophageal portosystemic collaterals [25]. By comparison, a CT scan can more comprehensively evaluate the anatomy and classification of SPSS, including paraesophageal varices or other perigastric collaterals.

### 6.1. Esophageal Varices

**Figure 3:**
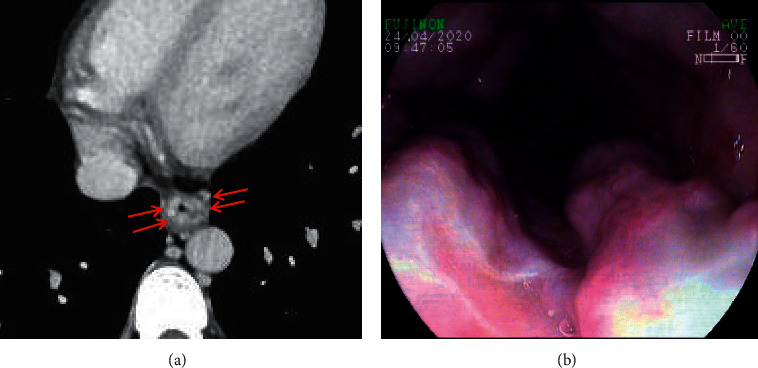
Esophageal varices. In a 38-year-old male with liver cirrhosis, the axial contrast-enhanced CT scan (a) on the portal vein phase demonstrated that esophageal varices (red arrow) were enhanced channels adjacent to the surface of the esophageal lumen, with round, tubular, or scalloped borders. Upper gastrointestinal endoscopy (b) showed several tortuous varices in the esophagus.

Esophageal varices, which refer to tortuously dilated esophageal veins [42], are one of the main complications of portal hypertension [43], and esophageal variceal bleeding is one of the most common causes of death in liver cirrhosis [44, 45]. About 30% of patients with liver cirrhosis develop esophageal variceal bleeding [42]. The mortality of each variceal bleeding episode is 10–20% [42]. On the portal vein phase of contrast-enhanced CT scan, esophageal varices manifest as enhanced channels adjacent to the surface of the esophageal lumen or protruding into the esophageal lumen, with round, tubular, or scalloped borders [13, 40] (Figure 3).

### 6.2. Paraesophageal Varices

**Figure 4:**
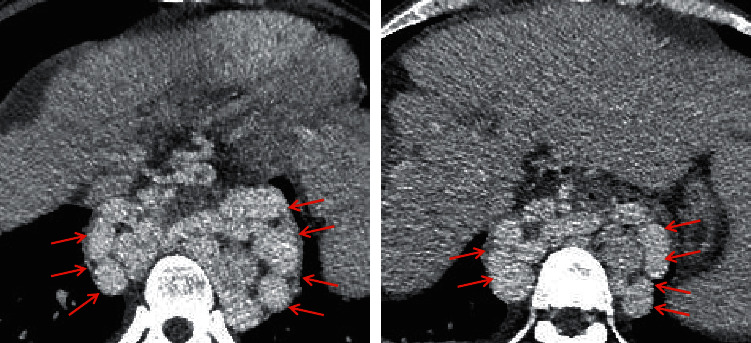
Paraesophageal varices. In a 37-year-old male with liver cirrhosis, the axial contrast-enhanced CT scan on the portal vein phase demonstrated that paraesophageal varices (red arrow) were tortuous vessels outside the esophageal wall.

Paraesophageal varices, which are dilated veins outside the esophageal wall [46], usually flow into the superior vena cava through the dilated azygos vein or hemiazygos vein and rarely into the inferior vena cava through the inferior phrenic vein [16, 47]. Paraesophageal varices can extend to the thoracic cavity, manifesting as a mass on the plain CT scan, which may be misdiagnosed as a mediastinal tumor [48, 49]. On the portal vein phase of contrast-enhanced CT scan, paraesophageal varices manifest as tortuous vessels outside the esophageal wall, reaching the level of aortic arch upwards and the cardia downwards [50] (Figure 4).

### 6.3. Gastric Varices

**Figure 5:**
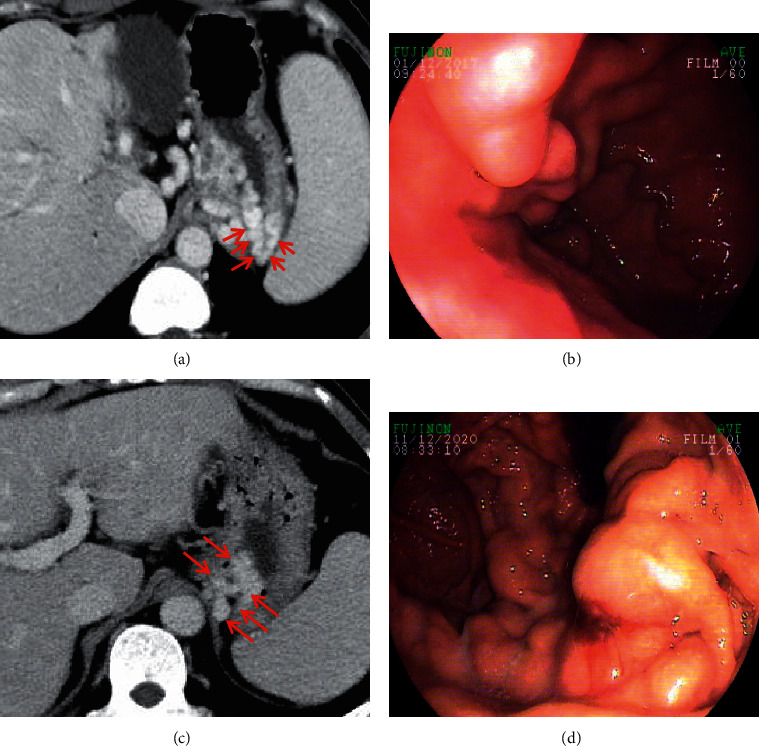
Gastric varices. In a 47-year-old female and a 56-year-old male with liver cirrhosis, the axial contrast-enhanced CT scan on the portal vein phase demonstrated that gastric varices (red arrow) were long, nodular, and tortuous enhanced channels at the gastric fundus. Upper gastrointestinal endoscopy showed dilated and tortuous varices at the gastric fundus. Note: (a, b) the contrast-enhanced CT scan and endoscopy of a 47-year-old female, respectively; (c, d) the contrast-enhanced CT scan and endoscopy of a 56-year-old male, respectively.

Gastric varices, which refer to tortuous and dilated veins located at the posterosuperior aspect of gastric fundus [46], are a major cause of upper gastrointestinal bleeding in patients with portal hypertension [51]. Gastric varices receive the blood from the left gastric vein, posterior gastric vein, and short gastric vein, and the blood flows into the lower esophageal vein, paraesophageal vein, and/or left inferior phrenic vein [52]. Esophageal and gastric varices are often concomitant [53, 54], which should be classified as GEVs, the most common type of gastric varices. Besides, isolated gastric varices (IGVs) are another type of gastric varices [17]. GEVs are further classified into two subtypes, including GEVs 1, which refer to esophageal varices extending along the lesser curvature of the stomach, and GEVs 2, which refer to esophageal varices extending along the gastric fundus [17]. IGVs are also further classified into two subtypes, including IGVs 1, which refer to varices located at the gastric fundus, and IGVs 2, which refer to isolated varices located at the gastric body and antrum or pylorus [17]. On the portal vein phase of the contrast-enhanced CT scan, gastric varices manifest as long, nodular, and/or tortuous enhanced structures [55] (Figure 5).

### 6.4. Left Gastric Vein

**Figure 6:**
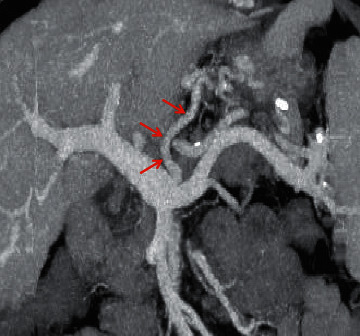
Left gastric vein. A 76-year-old female with a three-year history of autoimmune-related liver cirrhosis presented with recurrent hematemesis and melena. The coronal contrast-enhanced CT scan on the portal vein phase demonstrated the enlarged and tortuous left gastric vein (red arrow) at the lesser curvature of the stomach and the posterior wall of the left hepatic lobe.

The left gastric vein, also known as the gastric coronary vein [56], is divided into anterior and posterior branches [57]. The anterior branch forms a reticular vessel at the junction of the stomach and esophagus and anastomoses with the gastric varices, and the posterior branch anastomoses with the paraesophageal vein [57]. The anastomosis of the left gastric vein can be located at the main portal vein, splenic vein, splenoportal vein angle, or left portal vein branch [58]. Patients with the left gastric vein draining into the main portal vein are more prone to cause gastroesophageal variceal bleeding than those with the left gastric vein draining into the splenic vein or splenoportal vein junction [53]. On the portal vein phase of the contrast-enhanced CT scan, the enlarged and tortuous left gastric vein can be observed at the lesser curvature of the stomach and the posterior wall of the left hepatic lobe [59] (Figure 6).

### 6.5. Recanalized Paraumbilical Vein

**Figure 7:**
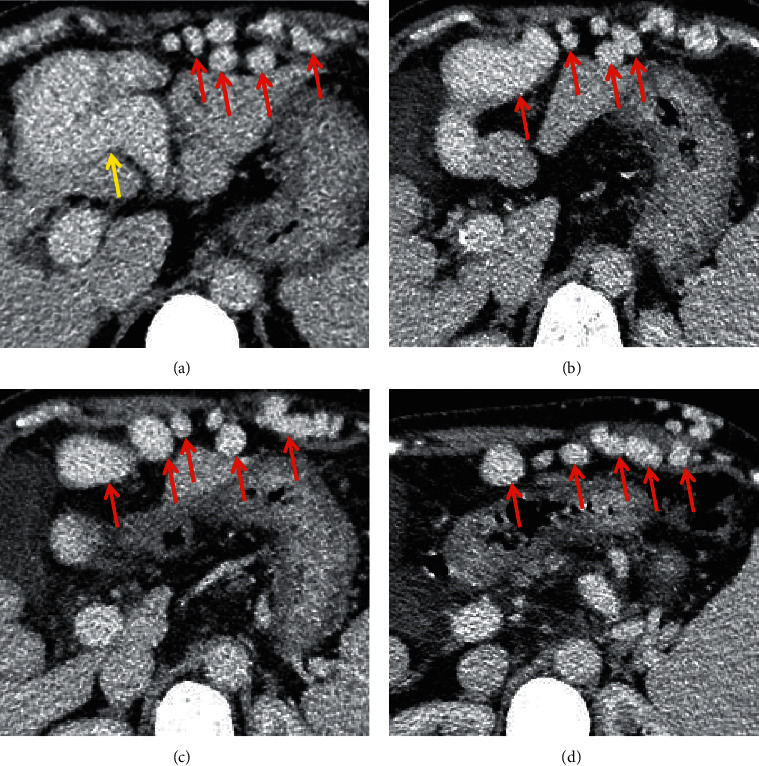
Recanalized paraumbilical vein. In a 65-year-old female with liver cirrhosis, the axial contrast-enhanced CT scan on the portal vein phase demonstrated that the recanalized paraumbilical vein (red arrow) was a round, dilated, or tubular enhanced structure, originating from the left portal vein branch (yellow arrow).

The paraumbilical vein, which originates from the left portal vein branch, can be reopened in the setting of portal hypertension and anastomoses with the abdominal wall vein [60]. The large paraumbilical vein can decrease the risk of esophageal variceal bleeding but precipitate the development of hepatic encephalopathy [61, 62]. On the portal vein phase of the contrast-enhanced CT scan, the recanalized paraumbilical vein is a round, dilated, or tubular enhanced structure, descending to the umbilicus from the left portal vein branch [63] (Figure 7).

### 6.6. Abdominal Wall Varices

**Figure 8:**
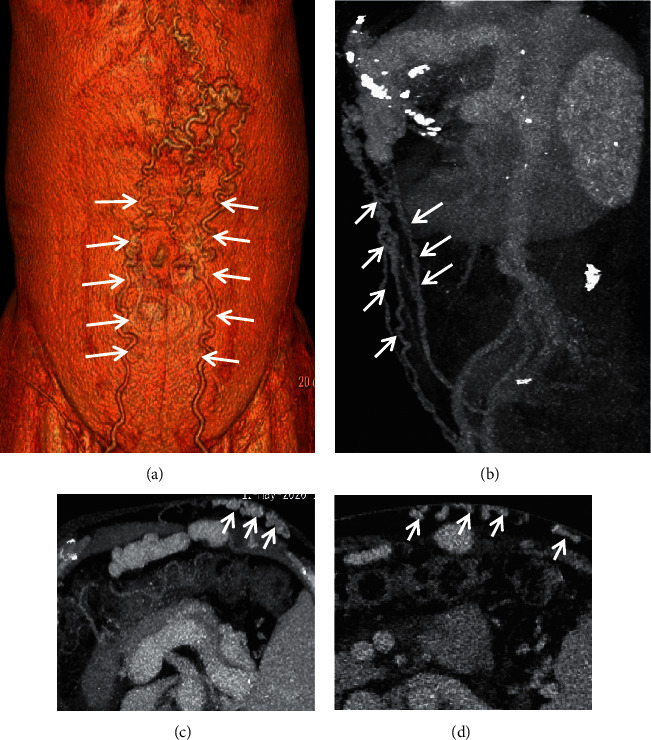
Abdominal wall varices. In a 65-year-old female with liver cirrhosis, the contrast-enhanced CT scan on the portal vein phase demonstrated that abdominal wall varices (white arrow) manifested as dilated, enhanced, and tortuous structures. (a) Three-dimensional reconstruction; (b) sagittal contrast-enhanced CT; (c, d) axial contrast-enhanced CT.

Abdominal wall varices, which refer to dilated or tortuous veins in the anterior abdominal wall, are commonly seen in the settings of portal hypertension or superior and inferior vena cava obstruction [64]. In patients with severe portal hypertension, varicose veins can be radiated from the umbilicus, which is called “caput medusa” sign [65]. When the superior vena cava is obstructed, the blood flow direction of superficial varicose veins above the umbilicus is downwards [66]. When the inferior vena cava is obstructed, the blood flow direction of superficial abdominal wall veins below the umbilicus is upwards [67]. The presence of abdominal wall varices predicts a worse survival of patients with liver cirrhosis [68]. On the portal vein phase of contrast-enhanced CT scan, abdominal wall varices manifest as dilated, enhanced, and tortuous structures [65] (Figure 8).

### 6.7. Spontaneous Splenorenal Shunt

**Figure 9:**
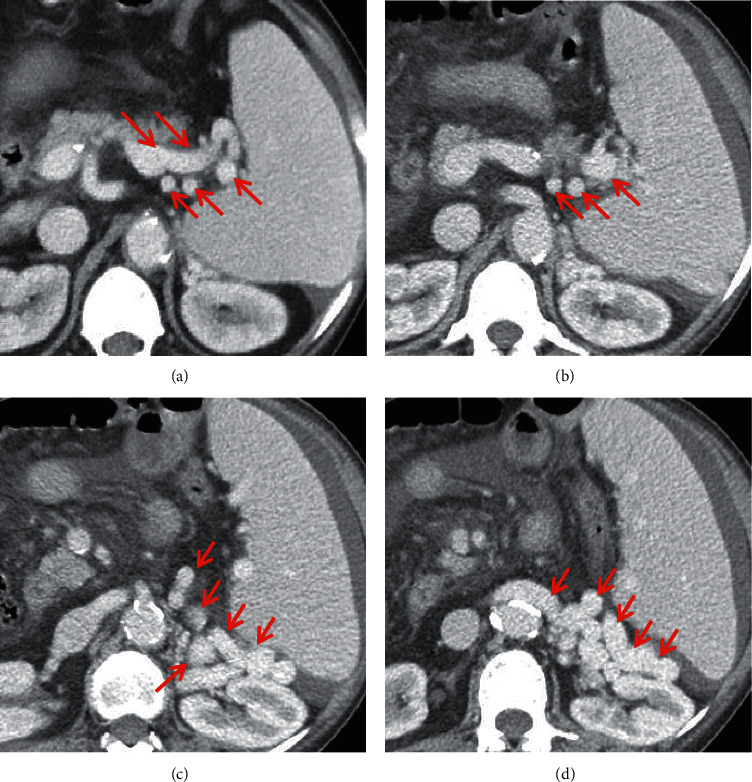
Splenorenal shunt. In a 62-year-old female with liver cirrhosis, the axial contrast-enhanced CT scan on the portal vein phase demonstrated that splenorenal shunt (red arrow) manifested as enhancement of abnormally dilated vessels originating from the splenic vein to the left renal vein.

**Figure 10:**
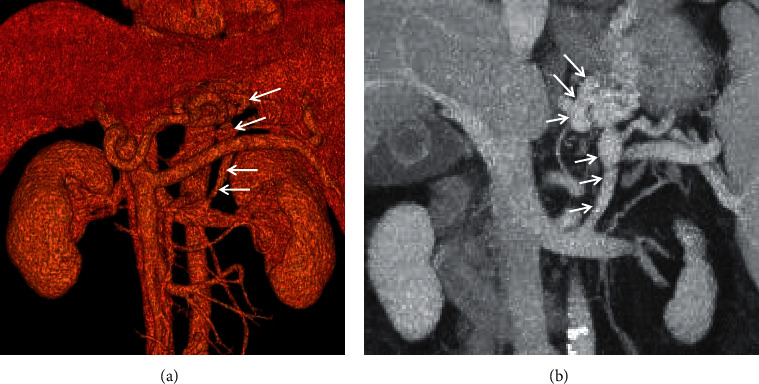
Gastrorenal shunt. A 62-year-old male with a four-year history of unexplained liver cirrhosis presented with recurrent hematemesis. The contrast-enhanced CT scan on the portal vein phase demonstrated that gastrorenal shunt (white arrow) manifested as enhancement of abnormally dilated vessels originating from the gastric vein to the left renal vein. (a) Three-dimensional reconstruction; (b) coronal contrast-enhanced CT.

SSRS often refers to abnormally dilated vessels connecting between the splenic vein and renal vein [14]. Besides, gastrorenal shunt, which refers to spontaneously abnormal communication between the gastric vein and renal vein [4], should be attributed to SSRS. SSRS may worsen liver function and increase the risk of hepatic encephalopathy and death in liver cirrhosis [9]. On the portal vein phase of contrast-enhanced CT scan, SSRS manifests as enhancement of abnormally dilated vessels originating from the splenic or gastric veins to the left renal vein [22] (Figures 9 and 10).

### 6.8. Retzius Vein

**Figure 11:**
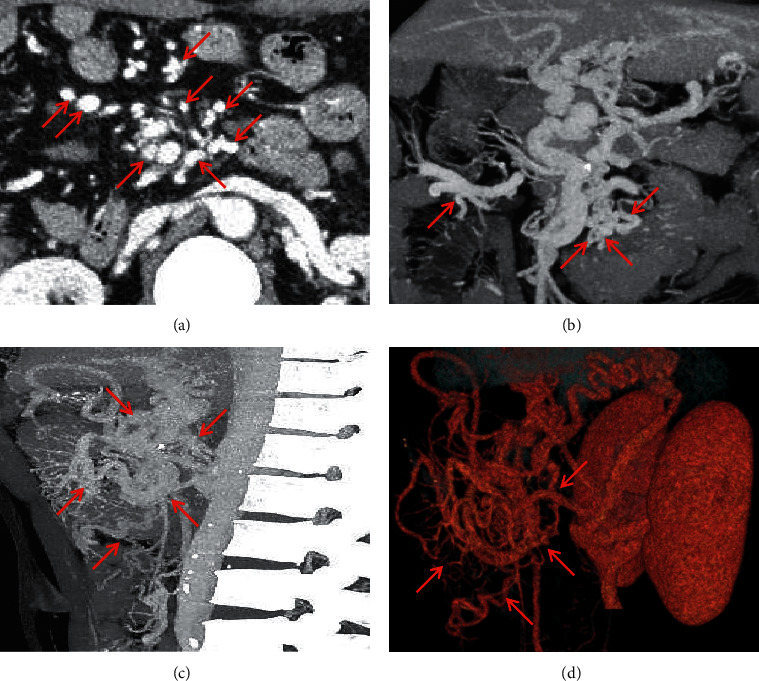
Retzius vein. A 48-year-old male patient with an eleven-year history of alcoholic-related liver cirrhosis presented with hematochezia. The contrast-enhanced CT scan on the portal vein phase demonstrated that the Retzius vein (red arrow) manifested as tortuously dilated vessels in the retroperitoneum in the portal vein phase. (a) Axial contrast-enhanced CT; (b) coronal contrast-enhanced CT; (c) sagittal contrast-enhanced CT; (d) three-dimensional reconstruction.

The Retzius vein refers to collateral vessels between the superior or inferior mesenteric vein and inferior vena cava in the retroperitoneum [47]. The Retzius vein is a number of small and easily neglected vessels in normal conditions and may gradually dilate when portal hypertension occurs. The blood of the Retzius vein mainly comes from the pancreaticoduodenal vein, ileocolic vein, lumbar vein, and gonadal vein [69]. On the portal vein phase of contrast-enhanced CT scan, the Retzius vein manifests as tortuously dilated vessels in the retroperitoneum (Figure 11).

### 6.9. Rectal Varices

Rectal varices, which refer to dilated rectal veins, originate from the anastomosis vessels of the superior rectal vein and the middle or inferior rectal veins in the lower rectum [70]. The inferior mesenteric vein is a feeding vessel for rectal varices, and rectal varices mainly flow into the internal iliac vein via the middle and/or inferior rectal vein [71]. Rectal variceal bleeding is rare, with an incidence of 0.45% to 3.6%, but it is often lethal [72]. On the portal vein phase of contrast-enhanced CT scan, rectal varices manifest as dilated and enhanced veins in the lower rectum.

## 7. Conclusions

SPSS mainly includes the esophagogastric venous plexus, paraumbilical venous plexus, retroperitoneal venous plexus, and rectal venous plexus, which manifests as tortuous, dilated, and enhanced vessels on the portal vein phase of contrast-enhanced CT scan. CT images are helpful for clinicians to diagnose different types of SPSS.

## Figures and Tables

**Table 1:** Comparison of decompensated events and death between with and without SPSS.

First author (year)	Study design	Enrollment period	Target population	No. of pts. in total	No. of pts. with shunt	Radiological evaluation	HE		Gastrointestinal bleeding		Ascites		Portal vein thrombosis		Death	
							With shunt	Without shunt	With shunt	Without shunt	With shunt	Without shunt	With shunt	Without shunt	With shunt	Without shunt
Qi et al. (2017) [20]	Cross-sectional	Jun. 2012–Dec. 2013	LC	105	11	CT or MRI	2 (18.2%)	4 (4.7%)	0 (0%)	17 (18.1%)	7 (63.6%)	52 (55.3%)	NA	NA	0 (0%)	4 (4.3%)
He et al. (2018) [21]	Cohort	Jan. 2004–Dec. 2014	LC	903	188	Angiography	89 (47.3%)	278 (39.0%)	175 (93.1%)	623 (87.1%)	126 (67.0%)	606 (84.7%)	NA	NA	68 (36.2%)	311 (43.5%)
Saks et al. (2018) [22]	Cohort	Jan. 2001–Feb. 2016	LT	741	173	CT or MRI	NA	NA	25%	28%	43%	59%	13%	4%	35%	42%
Simón-Talero et al. (2018) [7]	Cohort	2010–2015	LC	1729	1036	CT or MRI	L: 32%	8%	L: 25%	11%	L: 57%	32%	L: 18%	5%	L: 38%	32%
							S: 19%		S: 26%		S: 55%		S: 10%		S: 28%	
Allard et al. (2021) [23]	Cohort	Jan. 2003–Dec. 2016	LT	335	197	CT	NA	NA	NA	NA	NA	NA	36 (18.3%)	13 (9.5%)	7 (4.0%)	6 (4.9%)
Nardelli et al. (2021) [12]	Cohort	Mar. 2015–Jul. 2019	LC	222	141	CT	28 (20%)	11 (13%)	31 (22%)	13 (16%)	84 (60%)	47 (58%)	22 (16%)	3 (4%)	SPSS has a higher mortality	
Rathi et al. (2021) [24]	Cohort	2009–2017	LC	127	89	CT or MRI	Large SPSS had a higher risk of HE		NA	NA	12 (14%)	3 (8%)	27 (30%)	3 (8%)	NA	NA
Yi et al. (2021) [9]	Cohort	Dec. 2014–Aug. 2019	LC	122	37	CT or MRI	4 (10.8%)	6 (7.1%)	25 (67.6%)	67 (78.8%)	23 (62.2%)	50 (58.8%)	13 (35.1%)	27 (31.8%)	7 (18.9%)	4 (4.7%)
Dajti et al. (2022) [6]	Cohort	Jan. 2014–Dec. 2017	ACLD	235	141	CT or MRI	9 (6.4%)	3 (3.2%)	22 (15.6%)	5 (5.3%)	33 (23.4%)	13 (13.9%)	20 (14.2%)	3 (3.2%)	22 (15.6%)	9 (9.6%)

Pts., patients; LC, liver cirrhosis; LT, liver transplantation; SPSS, spontaneous portosystemic shunt; SSRS, spontaneous splenorenal shunt; HE, hepatic encephalopathy; CT, computed tomography; MRI, magnetic resonance imaging; L, large SPSS; S, small SPSS; ACLD, advanced chronic liver disease.
